# Supplementary material for: High-throughput atomistic modeling of nanocrystalline structure and mechanics of calcium aluminate silicate hydrate
Source: Nat Commun. 2025 Jun 19;16:5352. doi: 10.1038/s41467-025-60631-6 (PMC12179312; doi:10.1038/s41467-025-60631-6)
Supplement: Supplementary file 1 — Supplementary information [file 41467_2025_60631_MOESM1_ESM.docx]

Supplementary Information

High-throughput atomistic modeling of nanocrystalline structure and mechanics of calcium aluminate silicate hydrate

Yunjian Li^1^*, Cheng Chen^1^, Zhenning Li^2^, Zongjin Li^1^

^1^Faculty of Innovation Engineering, Macau University of Science and Technology, 999078, Macao

^2^State Key Laboratory of Internet of Things for Smart City, University of Macau, 999078, Macao

*Corresponding author: Yunjian Li: E-mail: liyunjian@must.edu.mo

1. **CASHgen nomenclature**

To use the CASHgen program, please refer to the instructions provided in the README file included with the code package, as well as in the GitHub repository：

<https://github.com/l98y3j26/CASHgen>

The following section explains the nomenclature (strings) used in the CASHgen program. The notation for each individual string is provided, along with its stoichiometry (Stoichio)) and atomic group charges in the block model.^1, 2, 3^

**Supplementary Table 1.** Dimmers.

| String | Brick model | Stoichio | Charge | Comment |
| --- | --- | --- | --- | --- |
| SL | < | CaSiO_4_ | -2 |  |
| BL | > | CaSiO_3_ | 0 |  |
| SR | < | CaSiO_3_ | 0 |  |
| BL | > | CaSiO_4_ | -2 |  |
| SLo | <' | CaSiO_3_OH | -1 | Protonated Oxygen |
| BLo | >' | CaSiO_2_OH | 1 |  |
| SRo | '< | CaSiO_2_OH | 1 |  |
| BLo | '> | CaSiO_3_OH | -1 |  |

**Supplementary Table 2.** Bridging sites.

| String | Brick model | Stoichio | Charge | Comment |
| --- | --- | --- | --- | --- |
| SU | S | SiO_2_ | 0 |  |
| SD | S | SiO_2_ | 0 |  |
| SUo | S' | SiOOH | 1 |  |
| SDo | S' | SiOOH | 1 |  |
| AUo | Al^IV^' | AlOOH | 0 |  |
| ADo | Al^IV^' | AlOOH | 0 |  |
| AUFo | Al^V^'' | AlO(OH)_2_ | -1 |  |
| ADFo | Al^V^'' | AlO(OH)_2_ | -1 |  |
| AUSo | Al^VI^'''' | Al(OH)_4_ | -1 |  |
| ADSo | Al^VI^'''' | Al(OH)_4_ | -1 |  |
| CU | C | Ca | 2 |  |
| CD | C | Ca | 2 |  |

**Supplementary Table 3.** Interlayer calcium or Aluminum.

| String | Brick model | Stoichio | Charge | Comment |
| --- | --- | --- | --- | --- |
| AIF | Al_IL_^V^'''' | AlO(OH)_5_ | -3 | Middle Interlayer |
| AIS | Al_IL_^VI^'''''' | Al(OH)_6_ | -3 |  |
| CII | C | Ca | 2 | Middle Interlayer |
| CIU |  |  |  | Behind Bridging |
| CID |  |  |  |  |
| XU |  |  |  | Pairing site |
| XD |  |  |  |  |

**Supplementary Table 4.** Hydroxide ions.

| String | Brick model | Stoichio | Charge | Comment |
| --- | --- | --- | --- | --- |
| oDL | o | OH | -1 | Interlayer |
| oDR |  |  |  |  |
| oUL |  |  |  |  |
| oUR |  |  |  |  |
| oXU |  |  |  | Bonded to XU/XD |
| oXD |  |  |  |  |
| oMDL |  |  |  | Bonded to dimmer Ca |
| oMDR |  |  |  |  |
| oMUL |  |  |  |  |
| oMUR |  |  |  |  |

**Supplementary Table 5.** Water.

| String | Brick model | Stoichio | Charge | Comment |
| --- | --- | --- | --- | --- |
| wDL | 1, 2, 3, … | H_2_O | 0 |  |
| wDR |  |  |  |  |
| wIL |  |  |  |  |
| wIR |  |  |  |  |
| wIR2 |  |  |  |  |
| wUL |  |  |  |  |
| wXU |  |  |  |  |
| wXD |  |  |  |  |
| W14 |  |  |  |  |
| W15 |  |  |  |  |
| W16 |  |  |  |  |
| wMUL |  |  |  | Bonded to dimmer Ca |
| wMDL |  |  |  |  |
| wMUR |  |  |  |  |
| wMDR |  |  |  |  |

**Upper Chain**

You can choose between option 1 and option 2, and for each option, you can only take one string for left, center, and right.

**Supplementary Table 6.** Possible configurations of the upper chain.

|  | Left | Center | Right |
| --- | --- | --- | --- |
| 1 | SL | SU | SR |
|  |  | SUo |  |
|  |  | AUo |  |
|  |  | AUFo |  |
|  |  | AUSo |  |
| 2 | SLo | CU | SRo |
|  | SL | Ø | SR |

**Below Chain**

You can choose between option 1 and option 2, and for each option, you can only take one string for left, center, and right.

**Supplementary Table 7.** Possible configurations of the below chain.

|  | Left | Center | Right |
| --- | --- | --- | --- |
| 1 | BL | SD | BR |
|  |  | SDo |  |
|  |  | ADo |  |
|  |  | ADFo |  |
|  |  | ADSo |  |
| 2 | BLo | CD | BRo |
|  | BL | Ø | BR |

**Interlayer**

Interlayers can contain any combination of interlayer calcium, interlayer aluminum, hydroxyl groups, and water, provided that overlap restrictions are considered. The table below summarizes the overlap between different configurations. Each row (1 to 9) lists strings where only one can be present in the unit cell at a time. Considering the possibility of interlayer aluminum, the following strings will not co-exist (“AIF”, “AIS”, “CII”, “ADo”, “ADFo”, “ADSo”, “SD”, “SDo”, “CD”, and “oDR”).

**Supplementary Table 8.** Overlaps in the interlayer.

|  | String1 | String2 | String3 | String4 | String5 | String6 | String7 |
| --- | --- | --- | --- | --- | --- | --- | --- |
| 1 | oMUR | wMUR | SU | SUo | AUo | AUFo | AUSo |
| 2 | oMDR | wMDR | SD | SDo | ADo | ADFo | ADSo |
| 3 | oUL | wUL | SU | SUo | AUo | AUFo | AUSo |
| 4 | oDR | wDR | SD | SDo | ADo | ADFo | ADSo |
| 5 | oMUL | wMUL |  |  |  |  |  |
| 6 | oMDL | wMDL |  |  |  |  |  |
| 7 | oUL | wUL |  |  |  |  |  |
| 8 | oDL | wDL |  |  |  |  |  |
| 9 | oXU | wXU |  |  |  |  |  |

1. **ERICA FF2 parameters**

**Van der Waal interactions^4^**

Oxygens: O_Si_ – Silicon Oxygen (Shell), O_w ­_– Water Oxygen, O_OH_ – Hydroxyl Oxygen

Hydrogens: H_w_ – Water Hydrogen, H_OH ­_– Hydroxyl Hydrogen

**Supplementary Table 9.** Overlaps in the interlayer.

| **Atom 1** | **Atom 2** | **Force Field** | **A [eV]** | **ρ [Å]** | **C [eV Å^6^]** | **Ref** |
| --- | --- | --- | --- | --- | --- | --- |
| Ca | O_si_ | Cement FF2 | 2152.36 | 0.309 | 0.09944 | ^5^ |
| Ca | O_w_ | Cement FF2 | 1286.60 | 0.297 | 0.00 |  |
| Ca | O_OH_ | Cement FF2 | 2251.05 | 0.297 | 0.00 |  |
| Si | O_Si_ | Cement FF2 | 1283.91 | 0.321 | 10.66 |  |
| Si | O_w_ | Cement FF2 | 1283.56 | 0.321 | 10.66 |  |
| Si | O_OH_ | Cement FF2 | 983.56 | 0.321 | 10.66 |  |
| O_si_ | O_Si_ | Cement FF2 | 22764.30 | 0.149 | 27.88 |  |
| O_Si_ | O_w_ | Cement FF2 | 22764.30 | 0.149 | 28.92 |  |
| O_Si_ | O_OH_ | Cement FF2 | 22764.00 | 0.149 | 13.94 |  |
| O_Si_ | H_w_ | Cement FF2 | 512.00 | 0.250 | 0.00 |  |
| O_OH_ | O_OH_ | Cement FF2 | 22764.30 | 0.149 | 6.97 |  |
| Al | O_Si_ | Cement FF2 | 1474.40 | 0.301 | 0.00 |  |
| Al | O_OH_ | Cement FF2 | 1032.00 | 0.301 | 0.00 |  |
| Al | O_w_ | Cement FF2 | 590.04 | 0.301 | 0.00 |  |

**Supplementary Table 10.** N-M Potential.

| **Atom 1** | **Atom 2** | **Force Field** | **E_0_ [eV]** | **r_0_ [Å]** | **n [-]** | **m [-]** | **Ref** |
| --- | --- | --- | --- | --- | --- | --- | --- |
| O_Si_ | H_OH_ | Cement FF2 | 0.0073 | 2.71 | 9 | 6 | ^5^ |
| O_w_ | O_OH_ | Cement FF2 | 0.0013 | 4.63 | 9 | 6 |  |
| O_w_ | H_OH_ | Cement FF2 | 0.0556 | 2.00 | 9 | 6 |  |
| O_OH_ | H_OH_ | Cement FF2 | 0.0073 | 2.71 | 9 | 6 |  |

**Supplementary Table 11.** L-J Potential.

| **Atom 1** | **Atom 2** | **Force Field** | **ε [eV]** | **σ [Å]** | **Ref** |
| --- | --- | --- | --- | --- | --- |
| O_w_ | O_w_ | SPC/Fw | 0.0067 | 3.16 | ^6^ |

**Supplementary Table 12.** Bond parameters.

| **Atom 1** | **Atom 2** | **Force Field** | **Bond type** | **Equation** | **Parameters** | **Ref** |
| --- | --- | --- | --- | --- | --- | --- |
| O_w_ | H_w_ | SPC/Fw | Harmonic | E2 | K = 22.96 eV  r_0_ = 1.012 Å | ^6^ |
| O_Si_Core_ | O_Si_Shell_ | Cement FF2 | Harmonic | E2 | K = 37.5 eV  r_0_ = 0 Å | ^5^ |
| O_OH_ | H_OH_ | Cement FF2 | Morse | E3 | D = 7.0525 eV  α = 3.1749 Å^-1^  r_0_ = 0.94285 Å | ^5^ |

**Supplementary Table 13.** Harmonic angle parameters.

| **Centre Atom** | **Atom 1** | **Atom 2** | **Force Field** | **E [eV]** | **ϴ_0_ [deg]** | **Ref** | |
| --- | --- | --- | --- | --- | --- | --- | --- |
| O_w_ | H_w_ | H_w_ | SPC/Fw | 1.64567 | 113.24 | ^6^ | |
| Si | O_OH_ | O_OH_ | Cement FF2 | 7.7482 | 109.47 | ^5^ | |
| Si | O_OH_ | O_Si_ |  |  |  |  |  |
| Si | O_Si_ | O_Si_ |  |  |  |  |  |
| O_OH_ | H_OH_ | Si | Cement FF2 | | 7.7482 | 141.50 | ^5^ |

1. **Equilibration of the C-A-S-H structure**

| 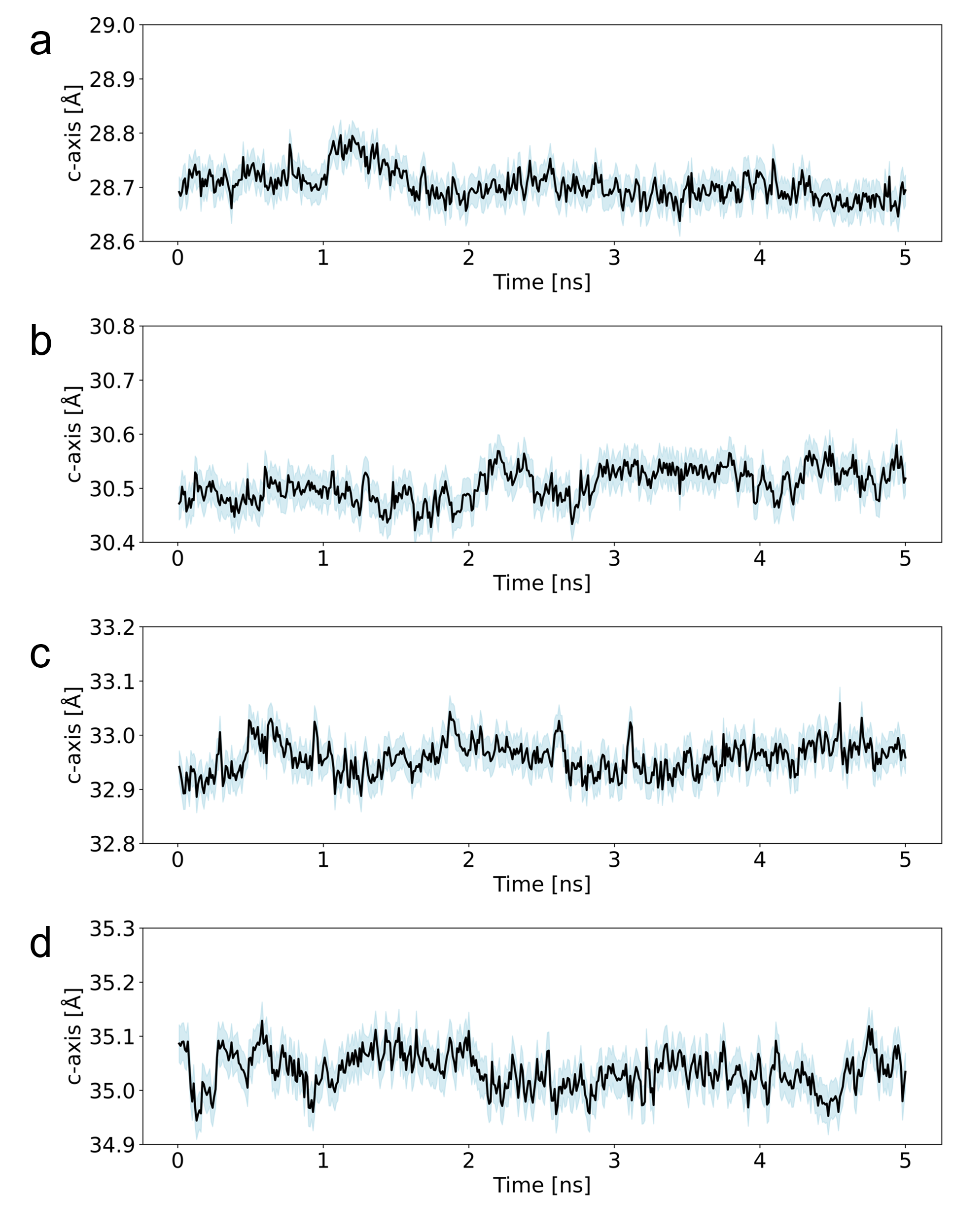 |
| --- |
| **Supplementary Figure 1.** The c-axis dimensions of the C-A-S-H structure with Al/Si = 0.15 were obtained after running a 5 ns NPT simulation. The Ca/Si ratios, from top to bottom, are **a** 1.3, **b** 1.5, **c** 1.7, and **d** 1.9, respectively. |

1. **Structural analysis**

|  |
| --- |
| **Supplementary Figure 2.** Layer spacings (z[Å]) as a function of 2H/Ca (H_2_O+2OH^-^)/Ca, * refers to the presence of Al(V) in the C-A-S-H structure. The square, diamond, triangle, and circle represent Ca/Si ratios of 1.3, 1.5, 1.7, and 1.9, respectively. |

|  |
| --- |
| **Supplementary Figure 3.** Layer spacings (z[Å]) as a function of 2H/Si (H_2_O+2OH^-^)/Si, * refers to the presence of Al(V) in the C-A-S-H structure. The square, diamond, triangle, and circle represent Ca/Si ratios of 1.3, 1.5, 1.7, and 1.9, respectively. |

|  |
| --- |
| **Supplementary Figure 4.** Ca^2+^/dimer as a function of MCL, * refers to the presence of Al(V) in the C-A-S-H structure. The square, diamond, triangle, and circle represent Ca/Si ratios of 1.3, 1.5, 1.7, and 1.9, respectively. |

|  |
| --- |
| **Supplementary Figure 5.** Ca^2+^ (bridging site)/dimer as a function of MCL, * refers to the presence of Al(V) in the C-A-S-H structure. The square, diamond, triangle, and circle represent Ca/Si ratios of 1.3, 1.5, 1.7, and 1.9, respectively. |

|  |
| --- |
| **Supplementary Figure 6.** Aluminum coordination number as a function of Ca/Si ratios, * refers to the presence of Al(V) in the C-A-S-H structure. The square, diamond, triangle, and circle represent Ca/Si ratios of 1.3, 1.5, 1.7, and 1.9, respectively. |

|  |
| --- |
| **Supplementary Figure 7.** Si-Si coordination number as a function of Ca/Si ratios, * refers to the presence of Al(V) in the C-A-S-H structure. The square, diamond, triangle, and circle represent Ca/Si ratios of 1.3, 1.5, 1.7, and 1.9, respectively. |

**Supplementary Table 14.** The coordination states and corresponding proportions of aluminates vary with Ca/Si ratios.

| Ca/Si | Al(IV)/% | Al(V) /% | Al(VI) /% |
| --- | --- | --- | --- |
| 1.3 | 50 | 25 | 25 |
| 1.5 | 25 | 25 | 50 |
| 1.7 | 0 | 25 | 75 |
| 1.9 | 0 | 25 | 75 |

**Supplementary Table 15.** The mean and standard deviation of the characteristics of the C-A-S-H atomic structures at given Ca/Si and Al/Si ratios.

| Ca/Si | Al/Si | MCL | 2H/Si | Si-OH/Si | Ca-OH/Ca |
| --- | --- | --- | --- | --- | --- |
| 1.3 | 0.05 | 5.11±0.73 | 1.21±0.03 | 0.27±0.03 | 0.26±0.04 |
|  | 0.10 | 6.96±1.16 | 1.24±0.03 | 0.27±0.02 | 0.23±0.04 |
|  | 0.15 | 9.73±1.63 | 1.27±0.03 | 0.27±0.03 | 0.20±0.03 |
|  | 0.15(interlayer) | 9.11±1.77 | 1.27±0.03 | 0.76±0.02 | 0.19±0.03 |
| 1.5 | 0.05 | 4.15±0.35 | 1.43±0.04 | 0.19±0.02 | 0.36±0.03 |
|  | 0.10 | 5.59±0.59 | 1.48±0.04 | 0.18±0.02 | 0.35±0.03 |
|  | 0.15 | 8.18±1.04 | 1.53±0.04 | 0.18±0.02 | 0.32±0.03 |
|  | 0.15(interlayer) | 7.75±1.16 | 1.54±0.04 | 0.19±0.02 | 0.32±0.03 |
| 1.7 | 0.05 | 3.87±0.29 | 1.77±0.05 | 0.08±0.02 | 0.46±0.03 |
|  | 0.10 | 5.11±0.40 | 1.84±0.04 | 0.09±0.02 | 0.44±0.02 |
|  | 0.15 | 7.69±0.93 | 1.92±0.04 | 0.09±0.02 | 0.42±0.03 |
|  | 0.15(interlayer) | 7.39±0.95 | 1.92±0.04 | 0.09±0.02 | 0.42±0.03 |
| 1.9 | 0.05 | 4.24±0.24 | 2.17±0.02 | 0.01±0.01 | 0.60±0.02 |
|  | 0.10 | 5.33±0.35 | 2.22±0.02 | 0.01±0.01 | 0.57±0.01 |
|  | 0.15 | 7.30±0.46 | 2.28±0.01 | 0.01±0.01 | 0.54±0.01 |
|  | 0.15(interlayer) | 6.82±0.52 | 2.28±0.01 | 0.01±0.01 | 0.53±0.01 |

**Supplementary Table 16.** The mean and standard deviation of Interlayer spacing(z), density, Ca^2+^/dimer and (H2O^+^ OH^−^）/Ca(2H/Ca) of the C-A-S-H structures predicted at given Ca/Si and Al/Si ratios. The Ca²⁺/dimer in this context refers to the number of Ca²⁺ ions that remain in the interlayer after all the vacant bridging sites in the C-A-S-H structure are filled by interlayer Ca ions.

| Ca/Si | Al/Si | z[Å] | Density | Ca2+/dimer | 2H/Ca |
| --- | --- | --- | --- | --- | --- |
| 1.3 | 0.05 | 12.64±0.30 | 2.38±0.03 | 0.61±0.10 | 0.93±0.02 |
|  | 0.10 | 12.76±0.30 | 2.39±0.02 | 0.72±0.09 | 0.95±0.02 |
|  | 0.15 | 12.80±0.33 | 2.41±0.03 | 0.79±0.11 | 0.97±0.01 |
|  | 0.15(interlayer) | 12.85±0.22 | 2.40±0.02 | 0.78±0.07 | 0.97±0.02 |
| 1.5 | 0.05 | 13.13±0.20 | 2.41±0.02 | 0.85±0.08 | 0.95±0.01 |
|  | 0.10 | 13.49±0.22 | 2.42±0.02 | 1.04±0.07 | 0.99±0.01 |
|  | 0.15 | 13.90±0.43 | 2.41±0.02 | 1.18±0.14 | 1.02±0.01 |
|  | 0.15(interlayer) | 13.80±0.29 | 2.41±0.02 | 1.14±0.11 | 1.03±0.01 |
| 1.7 | 0.05 | 14.25±0.24 | 2.40±0.01 | 1.22±0.10 | 1.05±0.01 |
|  | 0.10 | 14.46±0.25 | 2.41±0.02 | 1.34±0.07 | 1.09±0.01 |
|  | 0.15 | 14.98±0.26 | 2.41±0.01 | 1.54±0.09 | 1.13±0.01 |
|  | 0.15(interlayer) | 14.85±0.22 | 2.41±0.01 | 1.50±0.10 | 1.13±0.01 |
| 1.9 | 0.05 | 16.00±0.35 | 2.36±0.02 | 1.79±0.07 | 1.14±0.01 |
|  | 0.10 | 16.10±0.23 | 2.38±0.02 | 1.87±0.08 | 1.17±0.01 |
|  | 0.15 | 16.33±0.21 | 2.39±0.02 | 1.97±0.05 | 1.20±0.01 |
|  | 0.15(interlayer) | 16.28±0.21 | 2.38±0.02 | 1.93±0.06 | 1.21±0.01 |

**Supplementary Table 17.** The mean and standard deviation of calcium coordination number CN(Ca–O), aluminum coordination number CN(Al–O) of the C-A-S-H structures at given Ca/Si and Al/Si ratios. The "MD" refers to the relaxed structure obtained from the results, while "input" refers to the unrelaxed structure.

| Ca/Si | Al/Si | CN(Ca-O) | CN(Al-O) (MD) | | CN(Al-O) (input) |
| --- | --- | --- | --- | --- | --- |
| 1.3 | 0.05 | 6.23±0.04 | 4.83±0.21 | 4.90±0.14 | 4.75 |
|  | 0.10 | 6.27±0.05 | 4.85±0.08 |  |  |
|  | 0.15 | 6.32±0.06 | 4.97±0.10 |  |  |
|  | 0.15(interlayer) | 6.33±0.05 | 4.93±0.09 |  |  |
| 1.5 | 0.05 | 6.26±0.05 | 5.26±0.17 | 5.16±0.16 | 5.25 |
|  | 0.10 | 6.31±0.03 | 5.17±0.13 |  |  |
|  | 0.15 | 6.29±0.03 | 5.16±0.07 |  |  |
|  | 0.15(interlayer) | 6.31±0.04 | 5.17±0.14 |  |  |
| 1.7 | 0.05 | 6.30±0.05 | 5.44±0.16 | 5.50±0.13 | 5.75 |
|  | 0.10 | 6.34±0.03 | 5.52±0.15 |  |  |
|  | 0.15 | 6.39±0.03 | 5.54±0.07 |  |  |
|  | 0.15(interlayer) | 6.40±0.03 | 5.48±0.11 |  |  |
| 1.9 | 0.05 | 6.34±0.06 | 5.53±0.16 | 5.56±0.12 | 5.75 |
|  | 0.10 | 6.38±0.03 | 5.49±0.12 |  |  |
|  | 0.15 | 6.39±0.04 | 5.57±0.08 |  |  |
|  | 0.15(interlayer) | 6.39±0.03 | 5.63±0.07 |  |  |

|  |
| --- |

|  |
| --- |
| **Supplementary Figure 8.** Comparison of three individual PDFs and the mean PDF for Ca/Si = 1.5 and Al/Si = 0.15. |

|    |
| --- |
| **Supplementary Figure 9.** The mean pair distribution function (PDF) of bulk C-A-S-H structure at the given Ca/Si or Al/Si ratios. Different Al/Si ratios **a**  Ca/Si=1.3,1.5, **b** Ca/Si=1.7,1.9. ‘*’ refers to the presence of Al(V) in the C-A-S-H structure. |

|  |
| --- |
| **Supplementary Figure 10.** Mean Si-Si RDF at different Ca/Si ratios (Al/Si = 0.15). |

|  |
| --- |
| **Supplementary Figure 11.** Mean Si-Si RDF at different Al/Si ratios (Ca/Si = 1.7). |

|  |
| --- |
| **Supplementary Figure 12.** Comparison of MD predicted PDF with the experimental PDF of C-S-H with Ca/Si = 1. 7 from C.E. White et al. |

|  |
| --- |
| **Supplementary Figure 13.** Comparison of MD predicted PDF with the experimental PDF of C-A-S-H gel from C.E. White et al. |

|  |
| --- |
| **Supplementary Figure 14.** Comparison of MD predicted PDF with the experimental PDF from C.E. White et al. |

|  |
| --- |
| **Supplementary Figure 15.** Comparison of XRD experimental results and C-A-S-H model results. Experimental data for C/S1.2-A/S0.10 are from Wang J et al^7^., and experimental data for C/S1.5-A/S0.10 are from Zhu X et al^3^. |

1. **Mechanical Property Analysis**

| 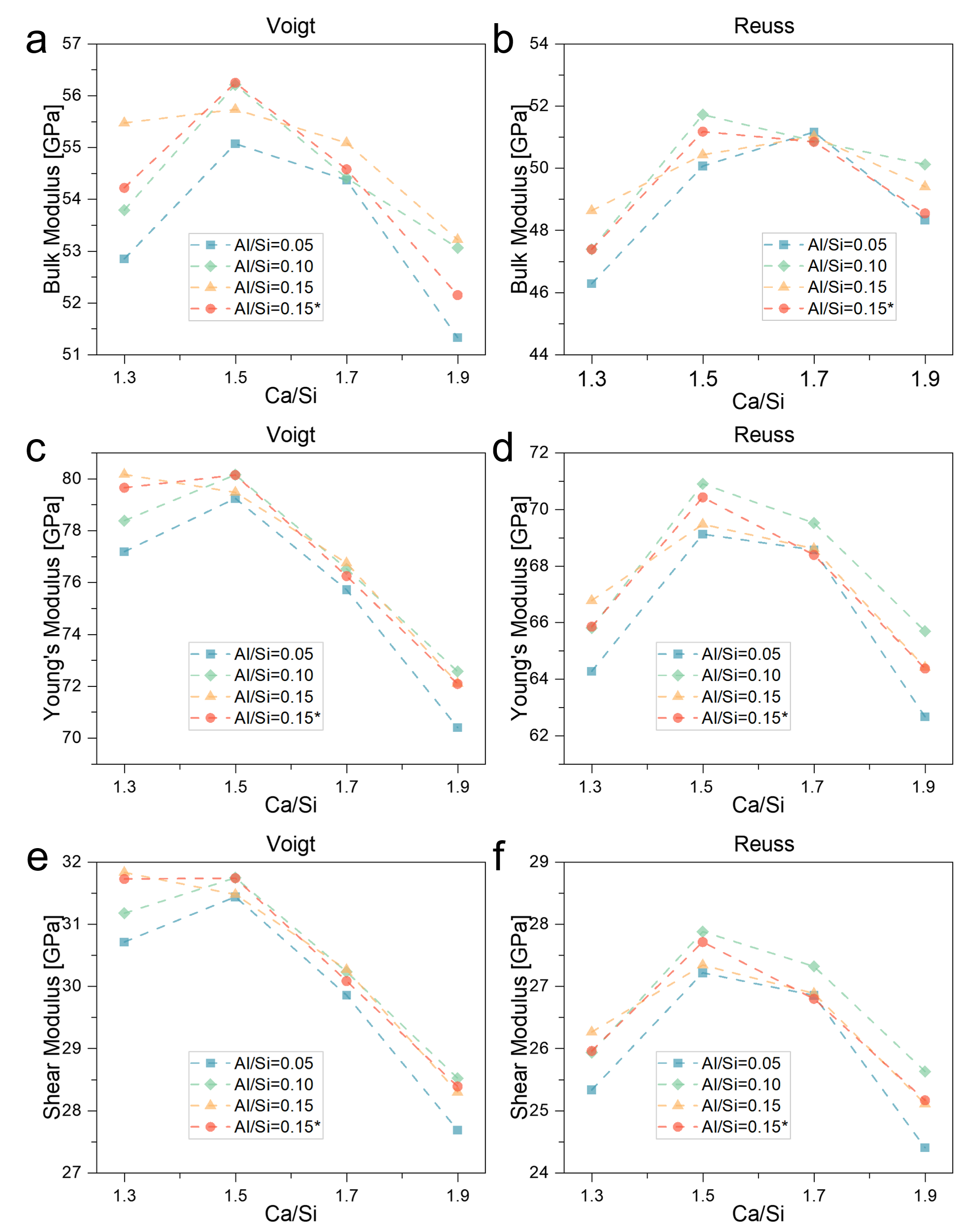 |
| --- |
| **Supplementary Figure 16.** The elastic moduli of C-A-S-H with different Ca/Si and Al/Si ratios. **a-b** The bulk Modulus averaging with Voigt and Reuss schemes. **c-d** The Young’s Modulus averaging with Voigt and Reuss schemes. **e-f** The shear Modulus averaging with Voigt and Reuss schemes. |

| 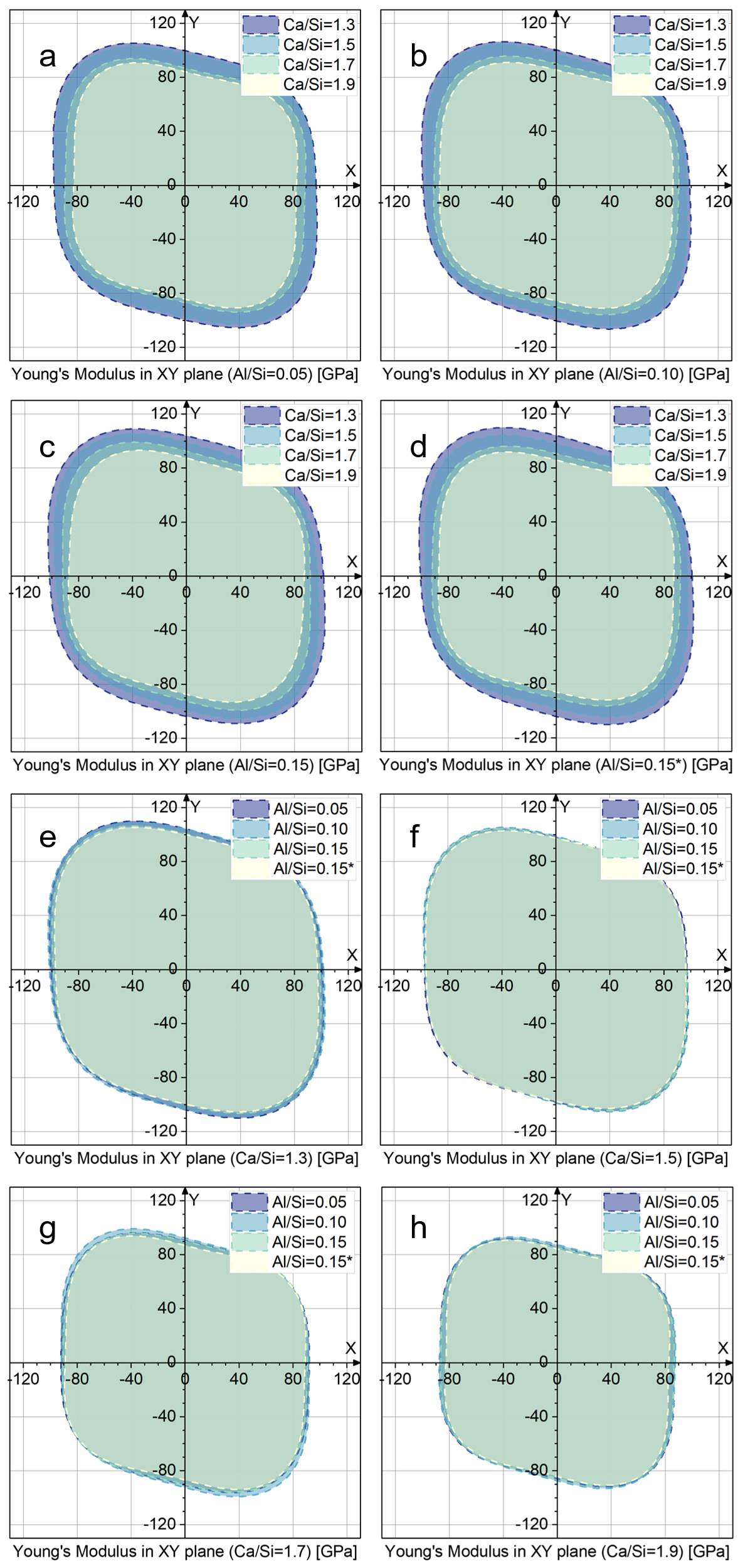 |
| --- |
| **Supplementary Figure 17.** Comparisons of the Young's modulus projections on the XY planes for same Al/Si= **a** 0.05, **b** 0.10, **c** 0.15, **d** 0.15* and different Ca/Si ratios, same Ca/Si= **e** 1.3, **f** 1.5, **g** 1.7, **h** 1.9 and different Al/Si ratios. ‘*’ refers to the presence of Al(V) in the C-A-S-H structure. |

| 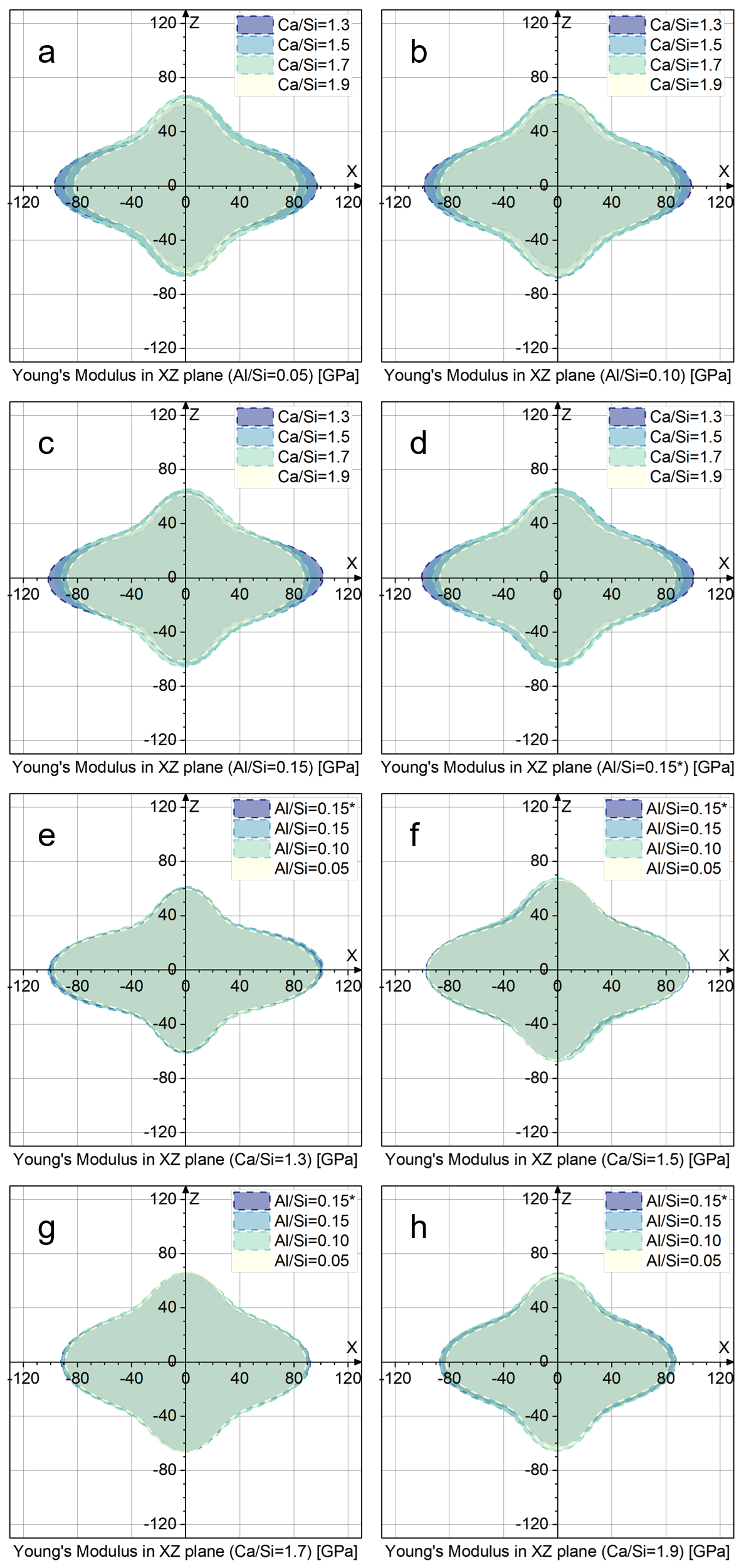 |
| --- |
| **Supplementary Figure 18.** Comparisons of the Young's modulus projections on the XZ planes for same Al/Si= **a** 0.05, **b** 0.10, **c** 0.15, **d** 0.15* and different Ca/Si ratios, same Ca/Si= **e** 1.3, **f** 1.5, **g** 1.7, **h** 1.9 and different Al/Si ratios. ‘*’ refers to the presence of Al(V) in the C-A-S-H structure. |

| 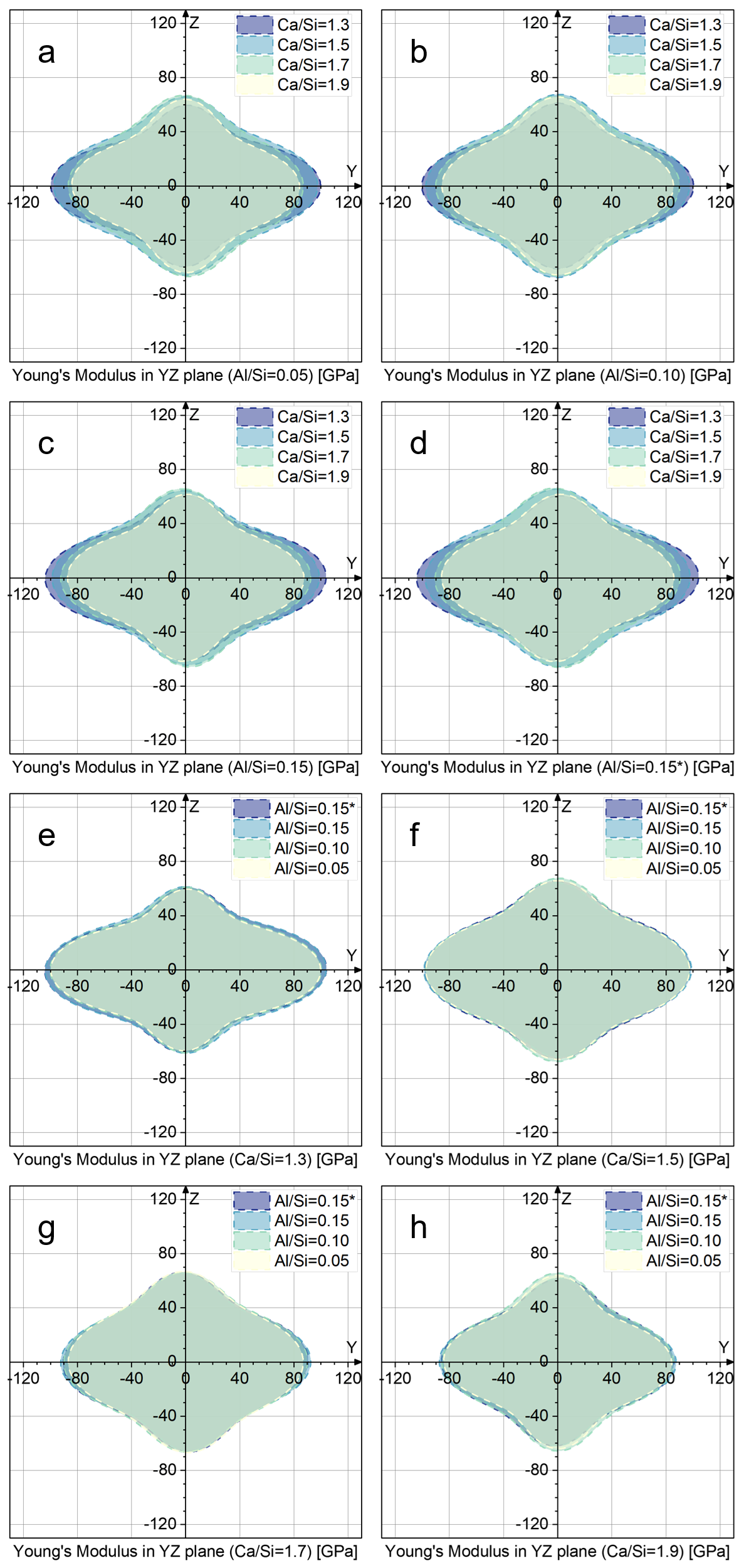 |
| --- |
| **Supplementary Figure 19.** Comparisons of the Young's modulus projections on the YZ planes for same Al/Si= **a** 0.05, **b** 0.10, **c** 0.15, **d** 0.15* and different Ca/Si ratios, same Ca/Si= **e** 1.3, **f** 1.5, **g** 1.7, **h** 1.9 and different Al/Si ratios. ‘*’ refers to the presence of Al(V) in the C-A-S-H structure. |

**Supplementary Table 18.** Elastic properties for C-A-S-H. K, E, and G stand for bulk, Young's, and shear modulus. * - C-A-S-H containing interlayer aluminum.

| Ca/Si | Al/Si | K[GPa] | G[GPa] | E[GPa] | H[GPa] | Poisson ratio (ν) |
| --- | --- | --- | --- | --- | --- | --- |
| 1.3 | 0.05 | 49.57 | 28.02 | 70.74 | 5.05 | 0.26 |
|  | 0.10 | 50.59 | 28.55 | 72.10 | 5.11 | 0.26 |
|  | 0.15 | 52.05 | 29.05 | 73.48 | 5.11 | 0.26 |
|  | 0.15* | 50.80 | 28.84 | 72.76 | 5.19 | 0.26 |
| 1.5 | 0.05 | 52.57 | 29.33 | 74.19 | 5.14 | 0.26 |
|  | 0.10 | 53.96 | 29.81 | 75.53 | 5.15 | 0.27 |
|  | 0.15 | 53.08 | 29.41 | 74.48 | 5.11 | 0.27 |
|  | 0.15* | 53.71 | 29.73 | 75.29 | 5.15 | 0.27 |
| 1.7 | 0.05 | 52.77 | 28.36 | 72.14 | 4.80 | 0.27 |
|  | 0.10 | 52.64 | 28.78 | 73.02 | 4.95 | 0.27 |
|  | 0.15 | 53.06 | 28.58 | 72.68 | 4.84 | 0.27 |
|  | 0.15* | 52.71 | 28.44 | 72.32 | 4.84 | 0.27 |
| 1.9 | 0.05 | 49.83 | 26.05 | 66.55 | 4.37 | 0.28 |
|  | 0.10 | 51.59 | 27.08 | 69.14 | 4.52 | 0.28 |
|  | 0.15 | 51.31 | 26.70 | 68.27 | 4.43 | 0.28 |
|  | 0.15* | 50.35 | 26.78 | 68.24 | 4.55 | 0.27 |

**Supplementary Table 19.** Elastic constants for different Ca/Si and Al/Si C-A-S-H models. * - C-A-S-H containing interlayer aluminum.

| **Ca/Si** | **1.3** | | | | **1.5** | | | | **1.7** | | | | **1.9** | | | |
| --- | --- | --- | --- | --- | --- | --- | --- | --- | --- | --- | --- | --- | --- | --- | --- | --- |
| **Al/Si** | **0.05** | **0.1** | **0.15** | **0.15*** | **0.05** | **0.1** | **0.15** | **0.15*** | **0.05** | **0.1** | **0.15** | **0.15*** | **0.05** | **0.1** | **0.15** | **0.15*** |
| **C11** | 116.53 | 118.20 | 121.51 | 119.48 | 116.32 | 117.46 | 117.31 | 118.19 | 110.45 | 110.97 | 112.19 | 112.10 | 102.50 | 106.89 | 108.19 | 107.18 |
| **C22** | 120.53 | 121.64 | 125.59 | 125.10 | 119.82 | 119.85 | 121.19 | 120.89 | 109.25 | 112.19 | 115.37 | 112.91 | 105.35 | 106.90 | 109.48 | 106.92 |
| **C33** | 65.02 | 66.87 | 67.67 | 66.40 | 72.89 | 75.43 | 72.05 | 73.25 | 75.79 | 75.06 | 74.37 | 74.80 | 72.26 | 74.69 | 70.63 | 70.28 |
| **C12** | 44.79 | 45.13 | 45.99 | 44.79 | 45.87 | 45.90 | 45.33 | 46.56 | 44.47 | 43.81 | 44.67 | 43.94 | 41.85 | 42.72 | 43.30 | 42.10 |
| **C13** | 20.68 | 21.08 | 22.35 | 21.09 | 22.62 | 24.92 | 24.29 | 24.27 | 25.85 | 25.04 | 25.05 | 24.64 | 24.35 | 25.62 | 25.71 | 24.82 |
| **C23** | 21.31 | 22.49 | 23.90 | 22.62 | 24.81 | 25.75 | 25.90 | 26.12 | 26.60 | 26.88 | 27.22 | 27.11 | 24.73 | 26.20 | 26.33 | 25.56 |
| **C44** | 18.84 | 19.45 | 20.30 | 20.11 | 21.75 | 21.83 | 21.62 | 22.22 | 21.50 | 21.33 | 20.48 | 21.18 | 18.52 | 19.74 | 18.84 | 19.55 |
| **C55** | 17.02 | 17.86 | 17.38 | 17.46 | 19.03 | 20.05 | 19.35 | 19.60 | 19.77 | 20.55 | 19.89 | 19.45 | 17.22 | 18.62 | 18.64 | 17.96 |
| **C66** | 45.94 | 45.89 | 47.30 | 46.91 | 44.51 | 44.79 | 44.74 | 45.08 | 41.83 | 41.79 | 42.63 | 41.76 | 39.64 | 39.61 | 39.68 | 40.47 |
| **C14** | -0.06 | 0.04 | 0.17 | 0.48 | 0.11 | -0.38 | -0.14 | -0.51 | 0.45 | -0.36 | -0.01 | -0.09 | 0.43 | 0.01 | -0.01 | -0.25 |
| **C15** | -0.32 | -0.25 | 1.05 | 0.19 | 0.42 | 0.53 | -0.27 | 0.59 | -0.05 | -0.07 | -0.05 | -0.39 | -0.22 | -0.30 | 0.41 | 0.51 |
| **C16** | -3.36 | -3.98 | -3.98 | -4.05 | -2.88 | -3.28 | -4.55 | -2.86 | -1.67 | -2.24 | -3.17 | -2.58 | -1.66 | -2.11 | -1.82 | -1.87 |
| **C24** | -0.04 | -0.12 | 0.48 | 0.99 | 0.01 | 0.04 | 0.05 | -0.01 | -0.08 | -0.01 | -0.11 | 0.33 | -0.42 | -0.09 | -0.16 | -0.19 |
| **C25** | -0.43 | 0.47 | 0.74 | 0.06 | 0.57 | 0.06 | -0.24 | 0.32 | 0.19 | 0.07 | 0.31 | -0.25 | -0.59 | -0.44 | 0.13 | 0.70 |
| **C26** | -6.70 | -7.12 | -6.90 | -7.51 | -6.22 | -7.46 | -8.10 | -6.86 | -6.16 | -6.31 | -7.28 | -6.51 | -6.20 | -5.90 | -6.15 | -5.92 |
| **C34** | 0.50 | 0.07 | -0.06 | 1.02 | -0.14 | 0.30 | -0.63 | -0.35 | -0.65 | -0.28 | -1.21 | -1.04 | -0.23 | -0.16 | 0.37 | 0.90 |
| **C35** | 0.03 | -0.09 | 0.45 | 0.00 | 1.62 | -0.06 | -0.29 | 0.94 | 0.59 | 0.23 | 0.90 | -0.08 | -0.16 | 0.17 | 0.28 | -0.34 |
| **C36** | -1.69 | -1.81 | -2.28 | -1.80 | -1.48 | -1.58 | -2.75 | -1.51 | -1.31 | -2.41 | -2.21 | -2.58 | -1.91 | -1.77 | -1.63 | -1.93 |
| **C45** | -1.98 | -2.75 | -2.65 | -3.52 | -3.11 | -2.59 | -3.08 | -2.81 | -1.78 | -1.83 | -2.09 | -2.49 | -1.40 | -1.38 | -2.19 | -1.61 |
| **C46** | -0.17 | -0.45 | -0.58 | -0.59 | -0.51 | -0.70 | -0.97 | -0.29 | -0.43 | -0.26 | -0.22 | -0.32 | -0.50 | -0.24 | -0.49 | -0.62 |
| **C56** | 4.73 | 4.63 | 4.63 | 4.54 | 4.32 | 4.50 | 3.88 | 4.43 | 4.46 | 3.87 | 3.60 | 3.68 | 3.16 | 3.11 | 2.75 | 3.17 |

**References**

1. Casar Z*, et al.* pyCSH: Automated atomic-level structure generation of bulk C-S-H and investigation of their intrinsic properties. *Cement and Concrete Research* **183**, 107593 (2024).

2. Kunhi Mohamed A, Parker SC, Bowen P, Galmarini S. An atomistic building block description of C-S-H - Towards a realistic C-S-H model. *Cement and Concrete Research* **107**, 221-235 (2018).

3. Zhu X*, et al.* Nature of aluminates in C-A-S-H: A cryogenic stability insight, an extension of DNA-code rule, and a general structural-chemical formula. *Cement and Concrete Research* **167**, 107131 (2023).

4. Valavi M, Casar Z, Kunhi Mohamed A, Bowen P, Galmarini S. Molecular dynamic simulations of cementitious systems using a newly developed force field suite ERICA FF. *Cement and Concrete Research* **154**, 106712 (2022).

5. Kunhi Mohamed A*, et al.* The Atomic-Level Structure of Cementitious Calcium Aluminate Silicate Hydrate. *Journal of the American Chemical Society* **142**, 11060-11071 (2020).

6. Wu Y, Tepper HL, Voth GA. Flexible simple point-charge water model with improved liquid-state properties. *J Chem Phys* **124**, 024503 (2006).

7. Wang J*, et al.* Effect of Ca/Si and Al/Si on micromechanical properties of C(-A)-S-H. *Cement and Concrete Research* **157**, 106811 (2022).
